# Credit risks in long-term care: a novel assessment of private long-term care institutions for sustainable development under the silver economy in China

**DOI:** 10.3389/fpubh.2026.1790055

**Published:** 2026-04-29

**Authors:** Zhouyi Gu, Andreea Claudia Serban, Jiayan Lv, Miltiadis D. Lytras, Pinqi Wang

**Affiliations:** 1 Universal Design Institute, College of Art and Design, Zhejiang Sci-Tech University, Hangzhou, China; 2 School of Information Technology, Zhejiang Financial College, Hangzhou, China; 3 Department of Economics and Economic Policy, Bucharest University of Economic Studies, Bucharest, Romania; 4 Library, Huzhou University, Huzhou, China; 5 School of Business and Economics, The American College of Greece, Athens, Greece; 6 Computer Science Department, College of Engineering, Effat University, Jeddah, Saudi Arabia

**Keywords:** credit risk assessment, machine learning, practical implication, private long-term care institutions, web based application

## Abstract

As private long-term care institutions play an increasingly important role in meeting long-term care needs in an aging society, assessing their credit risk is essential for supporting institutional sustainability, reducing information asymmetry, and strengthening risk-aware governance under the silver economy. However, existing studies have largely focused on subjective service-quality evaluations, with limited attention to objective, interpretable institution-level assessment based on publicly available data. Using publicly available records from 594 private long-term care institutions in Hangzhou, China, this study develops and validates an interpretable machine-learning framework for credit risk assessment. The framework integrates class-imbalance handling techniques with multiple machine-learning algorithms to improve predictive performance and identify the factors most closely associated with institutional creditworthiness. The results show that XGBoost with SMOTE achieves the best overall performance among the benchmark models across accuracy, sensitivity, specificity, and AUC. Feature importance and SHAP analyses identify four key predictors: registered capital and the number of tax-paying employees are positively associated with predicted creditworthiness, whereas financing history and patent counts are negatively associated with it in this setting. To enhance practical applicability, the final model is deployed in a Streamlit-based web application for real-time credit rating and visualization. Overall, this study extends credit risk assessment to the long-term care sector and provides an objective, interpretable, and deployable decision-support framework for families, managers, and regulators, with implications for strengthening the sustainable governance of private long-term care institutions.

## 1 Introduction

Population aging has become one of the most significant and irreversible demographic shifts of the 21st century (1). In this context, the ‘silver economy’ refers to the expanding set of markets and services designed to meet older adults’ needs, where institutional sustainability becomes a prerequisite for continuity of care. In China, this process is particularly pronounced. The proportion of individuals aged 60 and above increased from 10.5% in the Fifth National Population Census to 18.7% in the Seventh Census (2). According to the 2024 Bulletin on the Development of China’s Aging Undertaking, the number of people aged 60 and above has reached approximately 310 million, accounting for 22% of the total population (3). China has thus entered a moderately aged stage, creating far-reaching challenges for healthcare, long-term care, and social governance.

As aging accelerates, the health and functional status of older adults has intensified demand for long-term care (4). More than 75% of older adults have at least one chronic condition, and the prevalence of functional disabilities and cognitive impairment continues to rise (5). The World Health Organization projects that by 2050, a substantial proportion of China’s older population will require daily assistance (6). As traditional family-based caregiving becomes less viable, institutional long-term care services are playing an increasingly critical role in meeting older adults’ needs (7).

China’s long-term care service system currently comprises three major components: home-based care, community-based care, and institution-based care (8). Among these, institutional care—representing the professionalized segment of the long-term care service supply—has shown a rigidly increasing demand in meeting the care needs of older adults (9, 10). As an essential part of this system, private long-term care institutions play a crucial role in improving the quality of life and overall well-being of older adults (11). However, alongside the rapid expansion of such institutions, the sector faces multiple challenges, including uneven service content and quality, insufficient and inadequately trained staff, incomplete management structures, and low operational efficiency (12). Consequently, helping older adults and their families effectively identify and select high-quality private long-term care institutions has become a pressing issue of shared concern among scholars and practitioners.

Existing evaluations of long-term care institutions rely heavily on resident satisfaction surveys or qualitative assessments, which are inherently subjective and may underrepresent vulnerable groups such as those with cognitive impairments (13). Furthermore, such approaches often overlook institutional-level factors related to financial stability, innovation capacity, operational sustainability, and compliance risk. In a sector where continuity of care is essential, these limitations make it difficult for families and regulators to assess institutional reliability in a transparent and timely manner. Accordingly, institution-level assessment based on publicly available data is not only methodologically necessary but also practically important for reducing information asymmetry, supporting informed family choice, and strengthening risk-aware governance under the silver economy.

In the broader literature, credit risk assessment tools are widely used to evaluate the creditworthiness of individuals and micro and small enterprises (14, 15), and recent studies have shown that machine learning can substantially improve predictive performance in such tasks (16, 17). Given that many private long-term care institutions share key characteristics with micro and small enterprises, adapting credit risk assessment methods to this sector represents a promising avenue for evaluating institutional sustainability and safeguarding the welfare of older adults. However, systematic research on private long-term care institutions remains scarce. More importantly, few studies in this context simultaneously address three issues: objective institution-level risk assessment based on publicly available data, interpretable identification of key risk drivers, and practical deployment of the assessment framework for real-world decision support. This constitutes an important gap in the literature.

**Figure 1:**
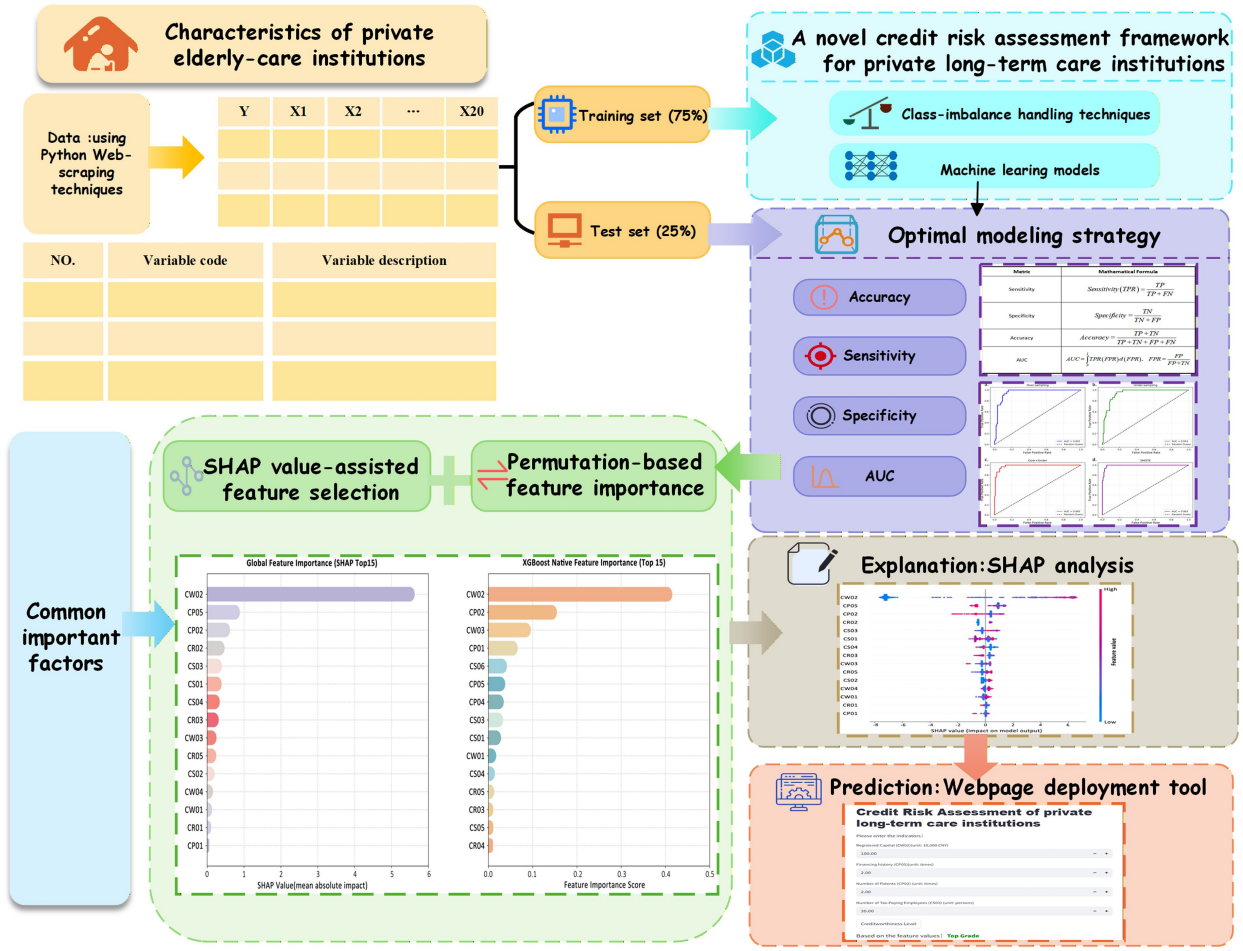
Road-map of the study (Source: Authors’ own).

The aim of this study is to develop and validate an interpretable machine-learning-based credit risk assessment framework for private long-term care institutions in China using publicly available institutional data. To achieve this aim, this study develops a credit risk assessment framework tailored to private long-term care institutions in China (see Figure 1). Publicly available institutional data were collected in a compliant manner and processed using resampling techniques to address class imbalance. Multiple machine learning algorithms were tested, with interpretability enhanced through feature importance and SHAP analysis. In addition, a web-based application was developed for real-time deployment, supporting both regulatory oversight and family decision-making.

This study makes three main contributions. First, it extends credit risk assessment to the long-term care sector, constructing a multidimensional dataset of 594 private institutions to capture financial strength, operational scale, innovation capacity, and compliance records. Second, it integrates class-imbalance handling with ensemble machine learning and interpretable AI techniques to improve predictive accuracy and transparency. Third, it bridges methodological innovation with practice by deploying a publicly accessible web tool, thereby enhancing institutional accountability and sustainable governance under the silver economy. In addition, the end-to-end pipeline (data preparation, resampling, model training, and explanation) is specified to facilitate replication and adaptation by local regulators.

The remainder of this paper is structured as follows. Section 2 reviews the relevant literature. Section 3 describes the data and methodology. Section 4 presents the empirical results. Section 5 offers the discussions, and Section 6 concludes the paper.

## 2 Related work

### 2.1 Population aging and the supply of long-term care services

Population aging is an irreversible global demographic trend (18). Since the beginning of the 21st century, the world’s population structure has been undergoing a profound transformation. According to projections by the World Health Organization, the number of people aged 60 and above will exceed 2.1 billion by 2050—more than double the figure in 2020 (1). As the world’s largest developing country, China faces a particularly prolonged and complex form of population aging, posing significant long-term challenges for social and economic development (2).

In terms of long-term care provision, China has established a three-tiered structure comprising home-based, community-based, and institution-based services (8). At present, there are just 20 long-term care institutions per 10,000 people aged 65 and above (9). However, as aging deepens and the numbers of empty-nest households, the oldest-old, and functionally impaired groups expand, long-term care demand shows a rigid upward trend (9, 10). Within this system, private long-term care institutions have grown rapidly but continue to face challenges such as uneven service quality, limited professional staffing, and low operational efficiency (12). Consequently, enabling older adults and their families to identify trustworthy and high-quality private institutions has become a pressing academic and policy concern.

### 2.2 Credit evaluation of private long-term care institutions

China’s institutional long-term care sector comprises public, private, and hybrid arrangements such as publicly constructed but privately operated facilities (5). In recent years, the number of private institutions has expanded significantly, driven by both demographic necessity and policy incentives. Within this context, credit evaluation offers a promising tool to support institutional sustainability and protect older adults from unreliable service providers (19).

Nevertheless, existing scholarship has paid limited attention to credit evaluation in this sector. Most studies rely on subjective indicators, such as resident satisfaction surveys or stakeholder interviews (20). While these approaches provide valuable insights, they underrepresent vulnerable groups such as cognitively impaired residents and often neglect institutional-level characteristics related to financial robustness, innovation capacity, or contractual performance (13). Although government agencies have introduced various service quality guidelines and conducted field-level assessments, there remains no unified national evaluation system for private long-term care institutions (21, 22). Furthermore, the data underpinning these evaluations are typically not transparent or accessible to potential service users and their families (23, 24). These limitations underscore the need for institution-level credit evaluations based on publicly available data.

### 2.3 Advances in credit risk assessment methodologies

Traditional credit risk assessment techniques have included statistical approaches such as univariate analysis, multiple discriminant analysis (MDA), and logistic regression (25–27). While widely used, these models impose restrictive statistical assumptions (e.g., variable independence, normality) and are less effective in dynamic, complex data environments (28).

With advances in big data and artificial intelligence, machine learning has become a mainstream alternative, offering nonlinear modeling capacity and superior predictive performance (15, 29). Empirical studies show that ensemble algorithms such as XGBoost, LightGBM, and Random Forest often outperform traditional models in prediction accuracy (30, 31). Some scholars also integrate machine learning with statistical evaluation methods to reduce subjectivity and enhance practical applicability (16, 32).

Despite their predictive advantages, machine learning models are often criticized for their “black-box” nature, which limits transparency and real-world adoption (33). In regulated or socially sensitive domains such as long-term care, explainability is critical for accountability, contestability of decisions, and managerial actionability (34). More recently, attention has turned to interpretable AI: SHAP and LIME provide transparent explanations by quantifying feature contributions to predictions, thereby addressing the “black-box” critique of machine learning (33, 35). These advances have been successfully applied in fields such as healthcare and small enterprise finance. However, their application to the long-term care sector remains underexplored.

### 2.4 Gaps in existing research and study contribution

Despite important progress in the literature, three key gaps remain. First, most long-term care evaluations emphasize subjective service quality while neglecting institutional-level indicators such as financial stability, operational scale, innovation capacity and compliance records. Second, although credit risk assessment methods have been widely applied to individuals and small enterprises, their adaptation to private long-term care institutions has been rare, leaving an important gap in understanding institutional sustainability. Third, while machine learning enhances predictive accuracy, limited research has combined it with interpretability tools to reveal the mechanisms driving institutional credit risk—an aspect crucial for regulators, managers, and families.

This study addresses these gaps in four ways. (1) It constructs a dataset of 594 private long-term care institutions using legally collected, publicly available data that capture multidimensional attributes beyond service quality. (2) It integrates multiple class-imbalance handling techniques with mainstream machine learning algorithms to develop a robust credit risk assessment framework. (3) It employs feature importance analysis and SHAP to identify and interpret the determinants of institutional creditworthiness. (4) It enhances real-world applicability by deploying a user-friendly web application, thereby bridging methodological innovation with governance practice.

## 3 Data and method

### 3.1 Data sources

Data on private long-term care institutions were collected from the Tianyancha platform^1^ in China using Python-based web-scraping techniques, in compliance with relevant legal and ethical requirements. The data collection was completed on November 30, 2025. Only publicly accessible institution-level records were used; no personal data were collected, and the crawler respected access-frequency limits to minimize platform burden. Using “long-term care services” as the search keyword, we obtained an initial sample of 715 institutions registered in Hangzhou, Zhejiang Province. During preprocessing, we removed only clearly invalid entries caused by scraping/parsing errors, inconsistent units, and duplicate records. The final analytic sample therefore contained 594 valid institutions.

The dataset was compiled from the Tianyancha platform using a non-subscribed user account, and only institution-level information that is publicly accessible after login was collected. It contains 21 variables. The dependent variable, Y_value (creditworthiness), is a binary indicator where 1 denotes high creditworthiness and 0 denotes low creditworthiness. Specifically, Y_value was constructed from the platform’s publicly disclosed credit-related indicator(s) and mapped into a binary outcome using a pre-defined threshold (according to the platform’s rating rule, institutions with a credit score of 80 or above were classified as high-creditworthiness and coded as 1, whereas those with a score below 80 were classified as low-creditworthiness and coded as 0), where 1 indicates institutions above the threshold and 0 otherwise. The remaining 20 explanatory variables capture financial, operational, innovation, and risk-related attributes. Table 1 provides detailed definitions.

**Table 1:** Description of variables in the credit risk dataset.

No.	Variable code	Variable description
1	CW01	Days since establishment
2	CW02	Registered capital
3	CW03	Stability of the legal representative
4	CW04	Number of shareholders
5	CS01	Enterprise size
6	CS02	Number of branches
7	CS03	Number of tax-paying employees
8	CS04	External investment capacity
9	CS05	Number of bids and tenders
10	CS06	Number of customers served
11	CP01	Qualification certificates
12	CP02	Number of patents
13	CP03	Job postings
14	CP04	Number of copyrights
15	CP05	Financing history
16	CR01	Associated risk alerts
17	CR02	Historical enforcement/penalty records
18	CR03	Associated abnormal-change monitoring
19	CR04	Number of judicial cases
20	CR05	Number of corporate data changes
21	Y_value	Creditworthiness (1 = high, 0 = low)

### 3.2 Class-imbalance algorithms

The dataset is highly imbalanced, with 482 low-creditworthiness institutions (81.1%) and only 112 high-creditworthiness institutions (18.9%). To address this imbalance, four resampling strategies were employed: random over-sampling, random under-sampling, combined over- and under-sampling, and the Synthetic Minority Over-Sampling Technique (SMOTE) (36). Random over-sampling increases minority representation by randomly duplicating minority instances, which may raise the risk of duplication-driven overfitting (36). Random under-sampling reduces majority dominance by randomly removing majority instances, but it may discard informative observations and lead to information loss (36). Combined over- and under-sampling provides a compromise by moderately increasing the minority class while moderately reducing the majority class, balancing class distribution while preserving information (36). SMOTE does not duplicate instances; instead, it generates synthetic minority samples by interpolating between minority observations and their nearest neighbors, expanding minority coverage in feature space and improving sample diversity (37, 38).

### 3.3 Model development and evaluation

The dataset was randomly divided into a training set (75%) and a test set (25%). The split was stratified to preserve the original class proportions across partitions. Six classifiers were applied: CART, C4.5, Random Forest (RF), Support Vector Machine (SVM), Bagging, and Extreme Gradient Boosting (XGBoost) (32). Hyperparameters for the six classifiers were optimized using grid search combined with manual fine-tuning under stratified five-fold cross-validation within the training set (39). To improve transparency and reproducibility, the final hyperparameter settings for all models are reported in Appendix Table A1. Final model performance was then evaluated on the held-out test set.

Several commonly used evaluation indexes were employed to assess model performance, including the area under the receiver-operating-characteristic (ROC) curve (AUC), sensitivity, specificity, and accuracy (33). Sensitivity reflects the model’s ability to correctly identify positive instances, whereas specificity reflects its ability to correctly identify negative instances. Accuracy summarizes the overall proportion of correctly classified samples, and AUC provides a global measure of discriminative performance across decision thresholds. To improve the robustness of performance reporting, these core test-set metrics are presented with 95% confidence intervals in Table 2. In addition, precision, F1 score, and the area under the precision-recall curve (PR-AUC) were calculated on the held-out test set and are reported in Appendix Table A2 to provide a more comprehensive evaluation under class imbalance.

**Table 2:** Classification results of different models under various sampling strategies.

Sampling strategy	Metric	CART	C4.5	RF	SVM	Bagging	Xgboost
Over-sampling	Sensitivity	0.793 (0.672–0.964)	0.759 (0.733–0.967)	0.931 (0.882–0.953)	0.724 (0.556–0.885)	0.862 (0.717–0.967)	0.931 (0.882–0.961)
	Specificity	0.825 (0.759–0.889)	0.808 (0.738–0.902)	0.850 (0.815–0.930)	0.808 (0.762–0.886)	0.833 (0.758–0.890)	0.883 (0.828–0.933)
	Accuracy	0.819 (0.768–0.879)	0.799 (0.735–0.833)	0.866 (0.839–0.940)	0.792 (0.745–0.863)	0.839 (0.772–0.893)	0.893 (0.852–0.940)
	AUC	0.809 (0.747–0.903)	0.783 (0.708–0.836)	0.946 (0.931–0.986)	0.889 (0.831–0.941)	0.928 (0.901–0.979)	0.955 (0.938–0.988)
Under-sampling	Sensitivity	0.793 (0.759–0.991)	0.793 (0.776–0.887)	0.931 (0.897–0.962)	0.828 (0.638–0.930)	0.862 (0.815–0.901)	0.862 (0.849–0.903)
	Specificity	0.833 (0.779–0.913)	0.817 (0.805–0.930)	0.817 (0.761–0.893)	0.742 (0.663–0.813)	0.825 (0.764–0.896)	0.875 (0.847–0.944)
	Accuracy	0.826 (0.799–0.913)	0.812 (0.706–0.883)	0.839 (0.809–0.913)	0.758 (0.678–0.819)	0.832 (0.792–0.906)	0.872 (0.866–0.953)
	AUC	0.813 (0.795–0.932)	0.805 (0.774–0.855)	0.932 (0.923–0.982)	0.880 (0.825–0.936)	0.922 (0.905–0.984)	0.943 (0.914–0.994)
Over + Under sampling	Sensitivity	0.832 (0.717–0.967)	0.862 (0.717–0.967)	0.966 (0.882–0.971)	0.793 (0.631–0.929)	0.872 (0.717–0.967)	0.966 (0.882–0.978)
	Specificity	0.818 (0.793–0.919)	0.858 (0.793–0.919)	0.875 (0.804–0.926)	0.792 (0.717–0.857)	0.878 (0.793–0.919)	0.917 (0.864–0.960)
	Accuracy	0.828 (0.798–0.906)	0.859 (0.798–0.906)	0.893 (0.838–0.932)	0.792 (0.724–0.852)	0.879 (0.798–0.906)	0.926 (0.879–0.959)
	AUC	0.860 (0.783–0.924)	0.860 (0.783–0.924)	0.974 (0.945–0.991)	0.888 (0.821–0.936)	0.933 (0.892–0.961)	0.983 (0.963–0.996)
SMOTE	Sensitivity	0.897 (0.759–0.912)	0.862 (0.826–0.923)	0.931 (0.821–0.956)	0.759 (0.595–0.911)	0.897 (0.759–0.901)	0.966 (0.882–0.975)
	Specificity	0.908 (0.853–0.951)	0.975 (0.949–0.987)	0.9420 (0.894–0.975)	0.858 (0.802–0.911)	0.908 (0.853–0.951)	0.950 (0.913–0.983)
	Accuracy	0.906 (0.859–0.946)	0.953 (0.939–0.993)	0.940 (0.899–0.973)	0.839 (0.785–0.893)	0.906 (0.859–0.946)	0.953 (0.919–0.979)
	AUC	0.902 (0.826–0.955)	0.919 (0.898–0.995)	0.983 (0.962–0.996)	0.888 (0.826–0.938)	0.957 (0.922–0.981)	0.993 (0.981–0.999)

Values are point estimates on the held-out test set; values in parentheses are 95% confidence intervals. AUC denotes the area under the receiver-operating-characteristic curve. The corresponding training-set performance for all models is reported in Appendix Table A3 to facilitate train-vs-test comparison. Source: Authors’ own computation.

### 3.4 Feature importance and interpretability

It is challenging to get the correct interpretation of a ML model. The SHAP method is an approach that could rank the importance of input features and explain the results of the prediction model, and it is implemented to overcome the “black-box” issue (40, 41).

The model’s permutation-based feature importance and SHAP value-assisted feature selection were used to rank important features and screen out 4 common important factors from the two methods. SHAP analysis was conducted on these 4 factors.

By comparing the two methods, four common important factors were identified: registered capital, number of patents, financing history, and number of tax-paying employees. SHAP further provided both global explanations— showing consistent feature attribution across the dataset— and local explanations, which interpret individual predictions. This dual interpretability framework helped reveal the mechanisms underlying institutional credit risk. These explanations describe how features contribute to the model’s predictions; they do not, by themselves, establish causality.

### 3.5 Webpage deployment tool based on streamlit framework

To bridge research and practice, the final optimized model (XGBoost with SMOTE) was deployed as a real-time web application using the Streamlit framework. The tool allows regulators, managers, and families to input institutional data and receive immediate predictions of creditworthiness. The application further categorizes institutions into four risk levels—Alert, Review, Watch, and Top Grade—thereby supporting regulatory oversight and informed family decision-making. The tool is intended as decision support rather than an automated decision-maker, and results should be interpreted alongside on-site inspections and qualitative assessments of care quality.

## 4 Results

### 4.1 Descriptive analysis

**Figure 2:**
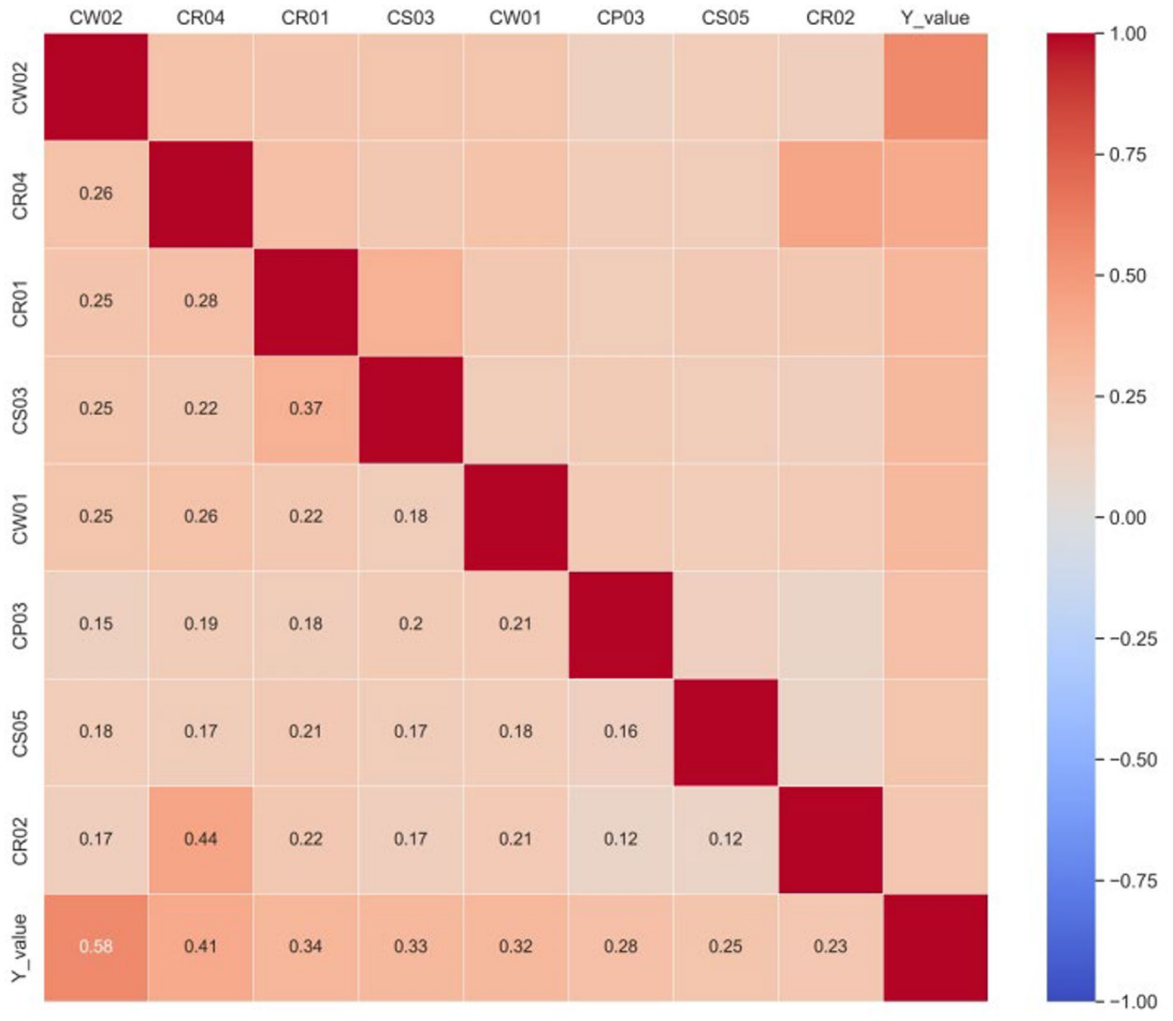
Heatmap of correlation coefficients for the eight explanatory variables most correlated with the response variable (Source: Authors own work using Python).

Among the 594 private long-term care institutions analyzed, 482 (81.1%) were classified as having low creditworthiness, whereas 112 (18.9%) were classified as having high creditworthiness, confirming a pronounced class imbalance. As shown in Figure 2, pairwise correlations remained low among the eight variables most strongly associated with the outcome. In addition, VIF diagnostics showed that all explanatory variables had values below 10, although three variables were slightly above 5, indicating only moderate collinearity rather than a level likely to compromise the subsequent machine learning analysis. Detailed VIF values are reported in Appendix Table A3.

### 4.2 Effects of class-imbalance handling

To mitigate imbalance, the training set was resampled using four strategies: random over-sampling, random under-sampling, combined over- and under-sampling, and SMOTE. Table 3 reports the resulting class distributions, showing that each method successfully balanced the dataset and thus improved the ability of classifiers to recognize minority-class institutions.

**Table 3:** Class-imbalance resampling schemes.

Sampling method	Dataset	Low creditworthiness *N* (%)	High creditworthiness *N* (%)	Total
Original data	Full sample	482 (81.14%)	112 (18.86%)	594
Simple random split	Training set	362 (81.35%)	83 (18.65%)	445
	Test set	120 (80.54%)	29 (19.46%)	149
Random over-sampling	New train 1	362 (50%)	362 (50%)	724
Random under-sampling	New train 2	83 (50%)	83 (50%)	166
Combined over- and under-sampling	New train 3	296 (46.69%)	338 (53.31%)	634
SMOTE	New train 4	362 (50%)	362 (50%)	724

Source: Authors’ own computation.

### 4.3 Model performance comparison

Six classifiers, namely CART, C4.5, RF, SVM, Bagging, and XGBoost, were trained under each resampling strategy. Their core test-set performance was evaluated using sensitivity, specificity, accuracy, and AUC, with 95% confidence intervals (32, 33), as shown in Table 2. Additional test-set metrics, including precision, F1 score, and PR-AUC, are reported in Appendix Table A2. Taken together, these results provide a more comprehensive assessment of model discrimination and classification quality under class imbalance. Table 2 summarizes the held-out test-set results, while the corresponding training-set performance is reported in Appendix Table A4 to facilitate train-vs.-test comparison.

Table 2 summarizes the test-set performance of models trained under four class-imbalance handling strategies, with hyperparameters tuned on the training set. First, there is clear variation across classifiers. Across all resampling strategies, the ensemble tree-based models (RF, Bagging, and especially XGBoost) generally outperform the single-tree methods (CART and C4.5) and SVM in terms of accuracy and AUC. This suggests that nonlinear relationships and complex interactions among institutional characteristics are better captured by ensemble approaches that aggregate multiple decision trees than by a single tree or a margin-based classifier. RF and XGBoost, in particular, achieve the highest AUC values under most settings, indicating stronger discriminative ability in separating high- and low-creditworthiness institutions.

Second, the choice of resampling strategy systematically affects model behavior. Compared with simple over-sampling or under-sampling, both the hybrid over + under sampling strategy and SMOTE lead to simultaneous improvements in sensitivity, specificity, and AUC for most classifiers. This indicates that these strategies are more effective in addressing class imbalance: the models become less biased toward the majority class and better able to correctly identify high-creditworthiness institutions without sacrificing performance on the low-creditworthiness group. SMOTE is especially effective because it synthesizes minority samples in feature space, thereby helping refine the decision boundary between the two classes.

Third, XGBoost exhibits strong and stable performance across all four resampling methods and performs best overall under SMOTE. Under this configuration, XGBoost attains a sensitivity of 0.966, correctly identifying the vast majority of high-creditworthiness institutions; a specificity of 0.950, accurately recognizing low-creditworthiness institutions; and an overall accuracy of 0.953. Although C4.5 under SMOTE reaches a comparable accuracy (0.953), its performance profile is more imbalanced, with very high specificity (0.975) but lower sensitivity (0.862). For credit risk evaluation and early warning, such imbalance is less desirable because it increases the likelihood that truly high-creditworthiness institutions are misclassified and may distort subsequent supervisory or investment decisions. By contrast, XGBoost under SMOTE offers a more favorable trade-off, delivering simultaneously high sensitivity and specificity and thus a more reliable classification pattern. The supplementary results reported in Appendix Table A2 are consistent with this conclusion, further confirming the superior overall performance of XGBoost under SMOTE.

**Figure 3:**
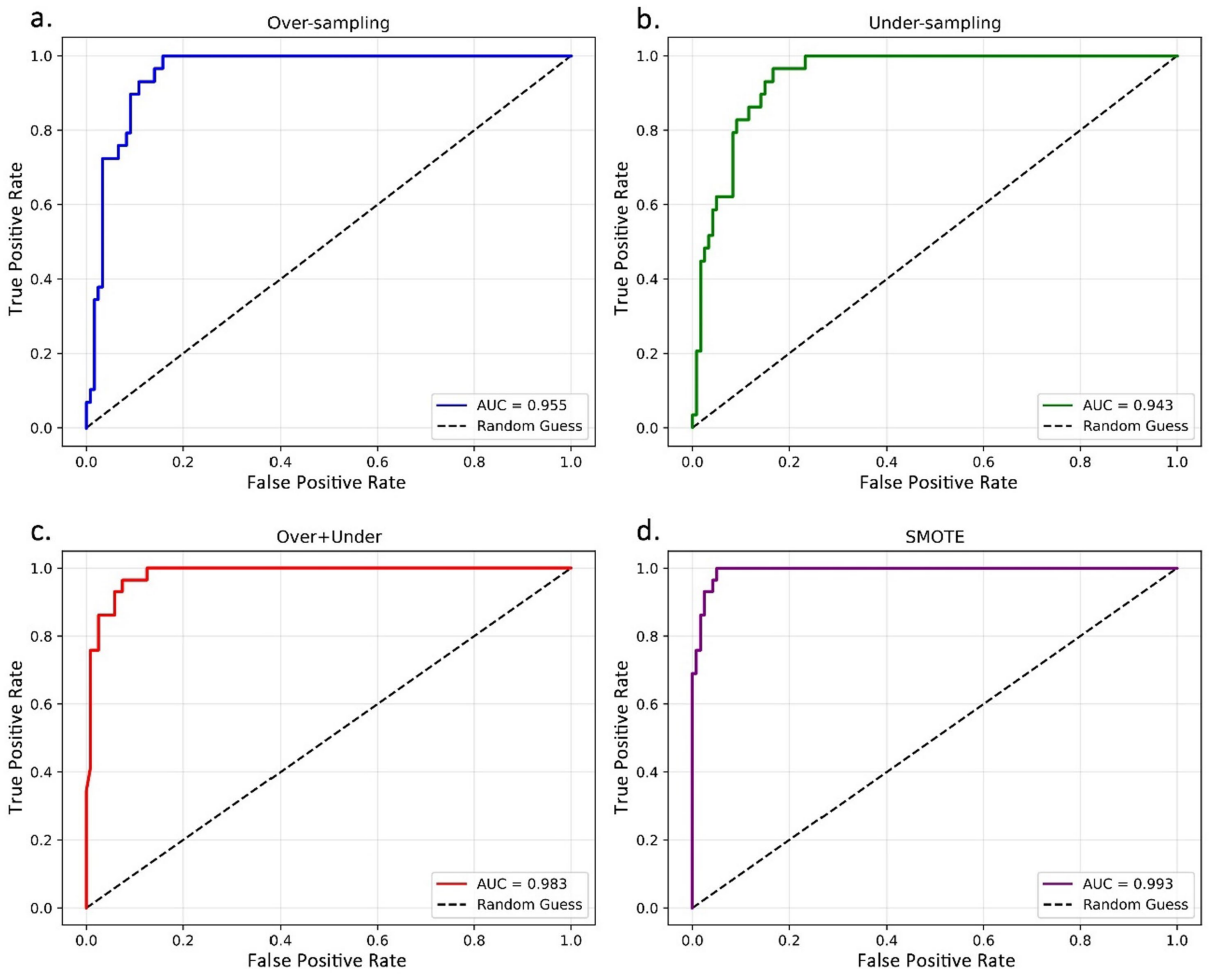
ROC curves of the XGBoost model under different sampling strategies (Source: Authors’ own using Python). **(a)** Over-sampling. **(b)** Under-sampling. **(c)** Over + under sampling. **(d)** SMOTE. The figure compares XGBoost trained with over-sampling, under-sampling, over + under sampling, and SMOTE. The SMOTE-based model achieves the highest AUC (0.993), with its curve closest to the upper-left corner, indicating the strongest discriminative performance among the four strategies.

Figure 3 visualizes the ROC curves of XGBoost under the four sampling strategies and further confirms the superiority of SMOTE. The SMOTE-based XGBoost model achieves an AUC of 0.993, exceeding the corresponding values under over-sampling (0.955), under-sampling (0.943), and the combined over + under sampling strategy (0.983). This result indicates that SMOTE enables XGBoost to achieve the most favorable balance between sensitivity and specificity across classification thresholds. Together with the supplementary precision, F1 score, and PR-AUC results reported in Appendix Table A3, these findings further support the robustness and practical value of the SMOTE-XGBoost combination for automated credit risk assessment in the long-term care sector.

### 4.4 Key feature analysis

**Figure 4:**
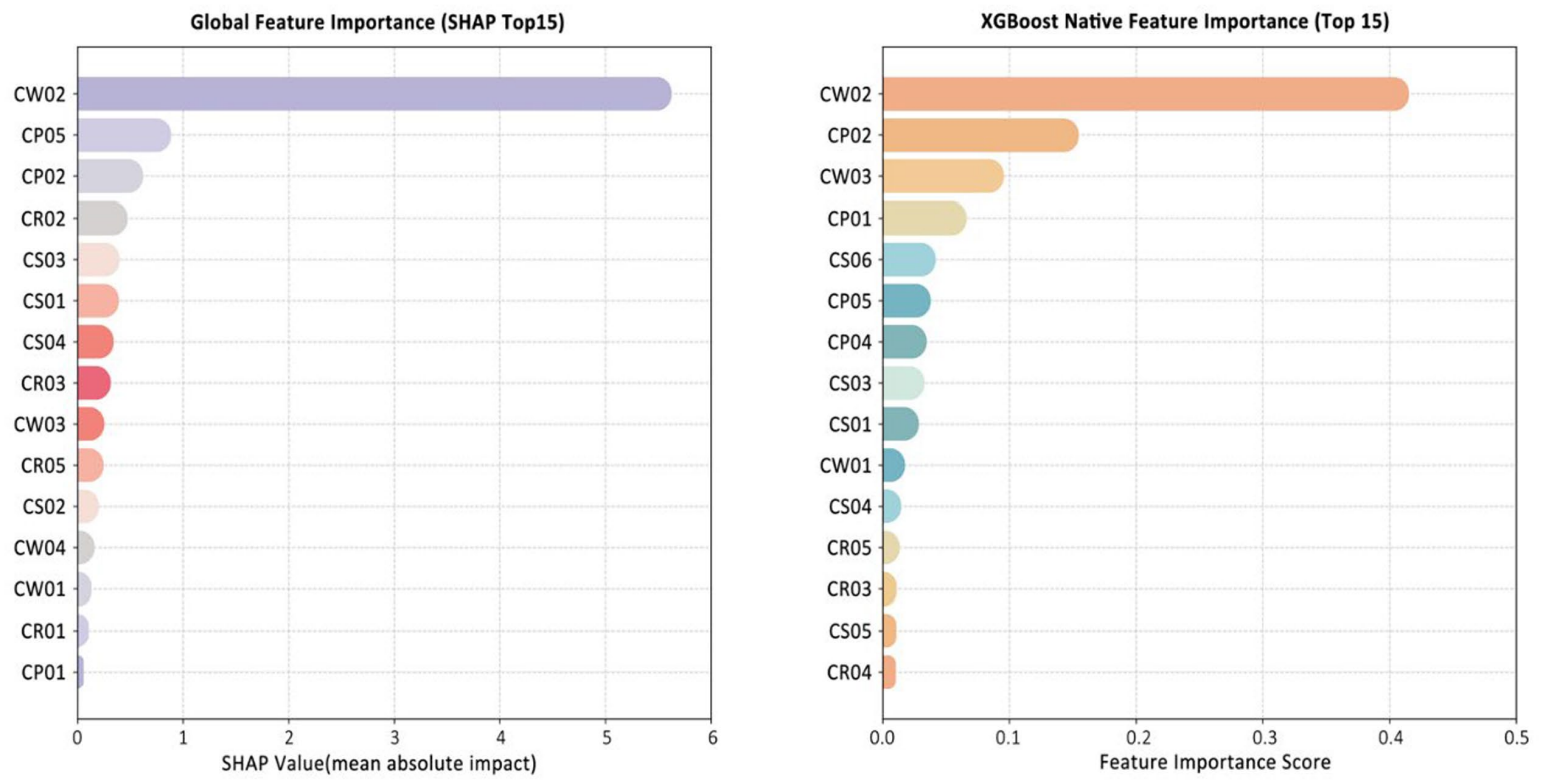
Top 15 feature importance rankings from XGBoost and SHAP (Source: Authors’ own computation).

To gain insight into the drivers of institutional creditworthiness, the best-performing model (XGBoost with SMOTE) was subjected to feature importance analysis using both SHAP values and the model’s native gain-based importance. Figure 4 reports the top 15 features ranked by each method. Although the detailed rankings differ slightly, four variables consistently stand out with markedly higher importance scores: registered capital (CW02), number of tax-paying employees (CS03), financing history (CP05), and number of patents (CP02). These variables were therefore selected as the core features for in-depth interpretation and for implementation in the web-based assessment tool.

**Figure 5:**
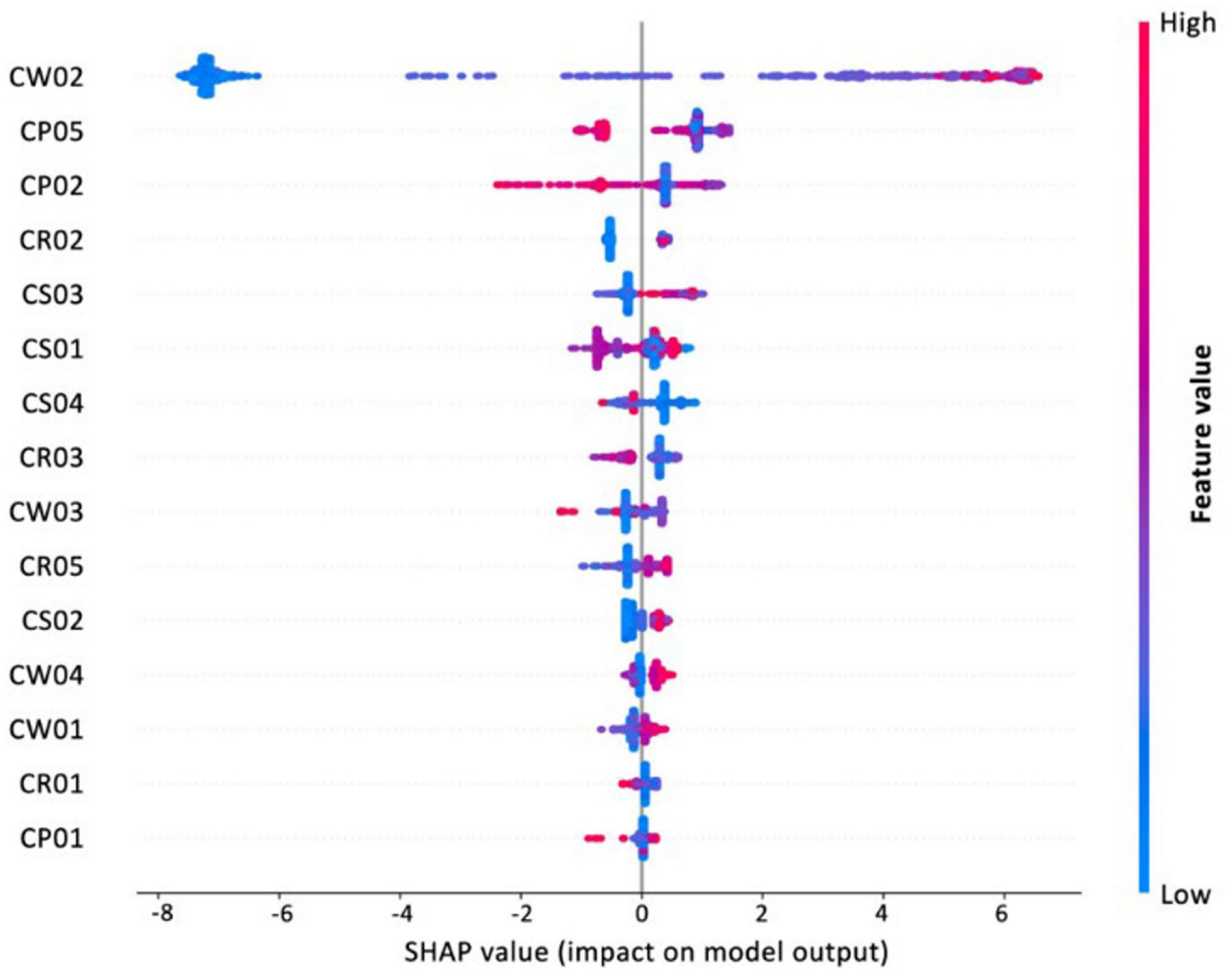
SHAP summary plot of the top 15 features, showing each feature’s overall impact on model prediction (Source: Authors’ own computation).

Figure 5 presents the SHAP summary plots. Registered capital and the number of tax-paying employees show a clear positive association with creditworthiness, indicating that higher levels of financial resources and workforce size enhance institutional stability and reduce risk. In contrast, both the number of patents and financing history exhibit negative SHAP values in certain cases—particularly at higher levels—implying that overly complex financing structures or excessive patenting may undermine institutional reliability. Taken together, these patterns point to a sector-specific “innovation–stability paradox,” in which aggressive expansion through financing or symbolic innovation can, paradoxically, compromise the long-term resilience of private long-term care institutions.

**Figure 6:**
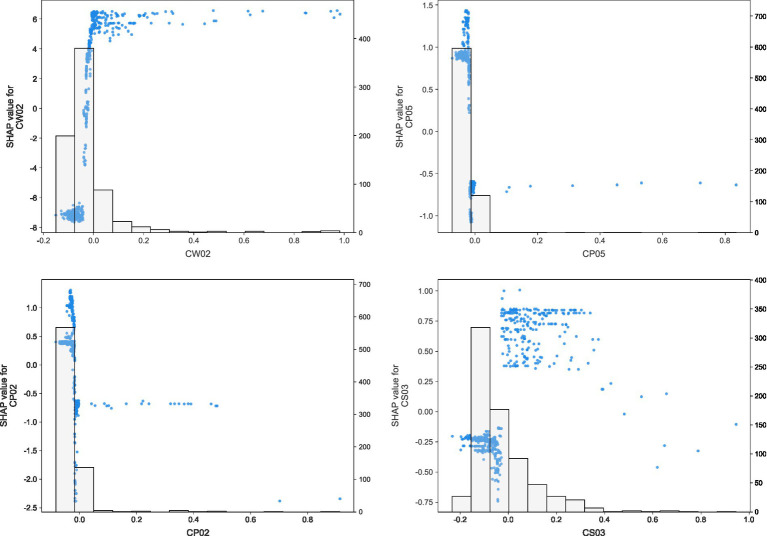
SHAP dependence plots for the four key features (Source: Authors’ own computation). SHAP dependence plots for registered capital (CW02), financing history (CP05), number of patents (CP02), and number of tax-paying employees (CS03). The plots illustrate the marginal contribution of each feature to the predicted creditworthiness of private long-term care institutions and reveal heterogeneous nonlinear effect patterns across the observed value ranges.

To further examine how these core variables affected model predictions, SHAP dependence plots were generated for registered capital (CW02), financing history (CP05), number of patents (CP02), and number of tax-paying employees (CS03), as shown in Figure 6. The plots reveal clear nonlinear effect patterns. Registered capital exhibited a predominantly positive contribution to predicted creditworthiness once it exceeded a low threshold, indicating that stronger capital reserves substantially improved institutional stability. By contrast, both financing history and number of patents showed generally negative marginal effects at higher values, suggesting that more frequent financing activities or excessive patent-related expansion may be associated with greater operational complexity and lower predicted creditworthiness. The dependence plot for the number of tax-paying employees indicates an overall positive association, although the magnitude of the contribution varies across the observed range, implying a nonlinear effect rather than a simple linear increase. These results provide more granular evidence for the interpretation derived from the SHAP summary plot and further support the existence of an innovation-stability trade-off in private long-term care institutions.

### 4.5 Convenient application for credit risk assessment

**Figure 7:**
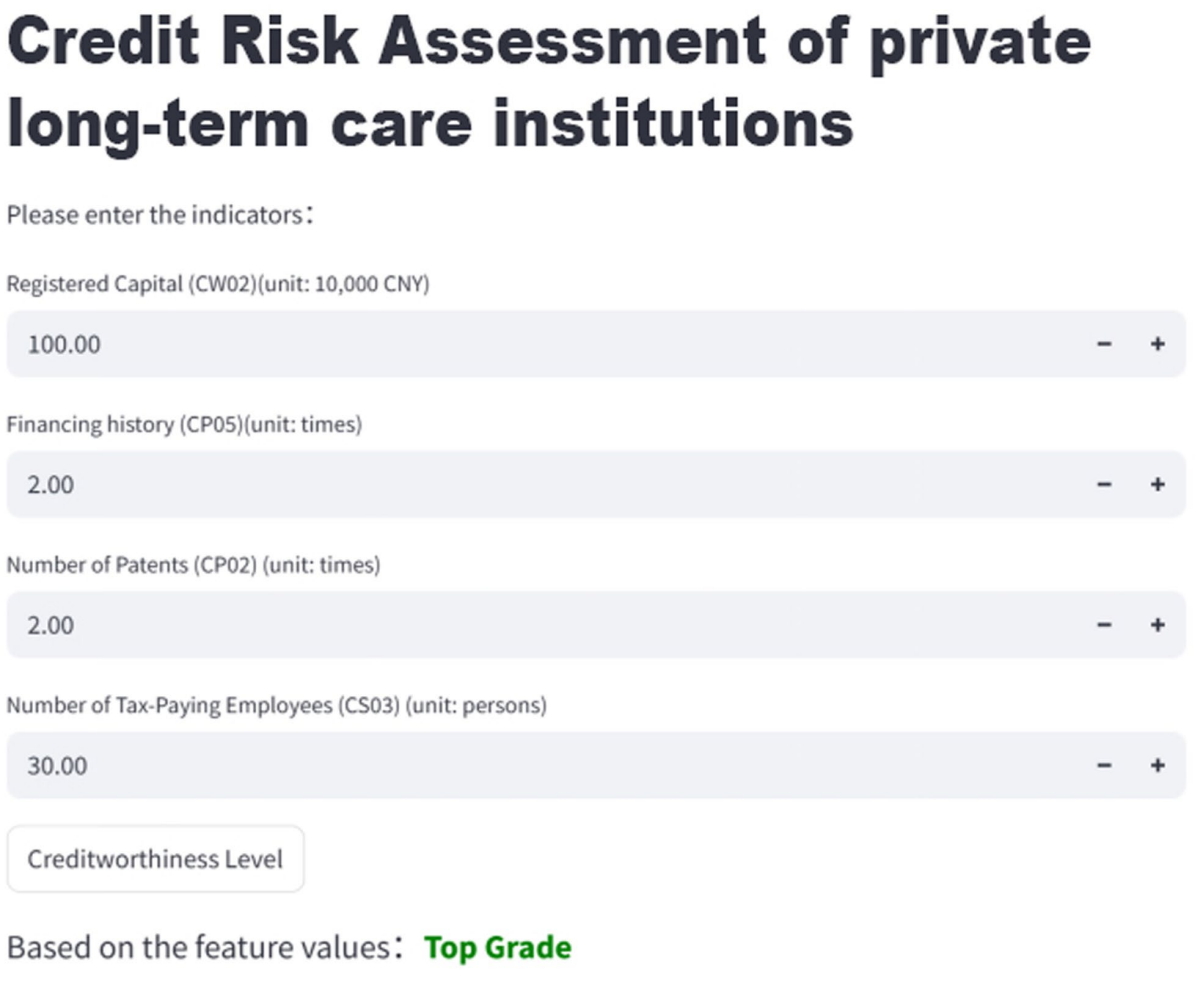
Convenient application for credit risk assessment (Source: Authors’ own computation).

To enhance practical utility, the optimized SMOTE- XGBoost model was deployed as a real-time web application using the Streamlit framework. As shown in Figure 7, the interface allows users to input institutional values for the four key features and instantly obtain a predicted creditworthiness level.

Institutions are categorized into four risk levels: (1) Alert: highest risk, requiring immediate attention; (2) Review: medium risk, requiring further evaluation; (3) Watch: relatively low risk, but continued monitoring is advised; (4) Top Grade: strong credit performance and reliability. This user-friendly tool supports regulators in targeted supervision, assists managers in self-assessment, and empowers families to make informed decisions. The web application is publicly accessible at https://english-str-gxatugjtgzy62ccjc92cmg.streamlit.app/.

## 5 Discussion and implications

### 5.1 Main findings

This study developed and validated a credit risk assessment framework specifically tailored to private long-term care institutions in China, where transparent and objective evaluation tools remain limited. By integrating resampling techniques with multiple machine learning models, the framework was able to handle pronounced class imbalance while maintaining both high predictive accuracy and interpretability. Across all imbalance-handling strategies, the ensemble tree-based methods, especially XGBoost, consistently outperformed single-tree models and SVM, indicating that institutional credit risk in this context is shaped by nonlinear relationships and complex interactions among features rather than by simple linear effects. Under the SMOTE resampling strategy in particular, XGBoost achieved a sensitivity of 0.966, a specificity of 0.950, an accuracy of 0.953, and an AUC of 0.993. This balanced performance profile—simultaneously high sensitivity and specificity—suggests that the model can effectively distinguish high-creditworthiness institutions from low-creditworthiness ones, avoiding an overemphasis on either false alarms or missed risks. These results confirm that the proposed framework is well suited for automated credit risk evaluation and early warning in the long-term care sector.

Beyond classification accuracy, the model yields substantive insights into the underlying drivers of institutional creditworthiness. Feature importance and SHAP analysis jointly identified four key determinants: registered capital, workforce size, financing history, and patent counts. Registered capital and the number of tax-paying employees exert stable, positive effects on predicted creditworthiness, underscoring the foundational role of financial robustness and organizational scale in sustaining institutional credibility. Institutions that are better capitalized and employ a larger, formally registered workforce are more likely to maintain stable operations, fulfill contractual obligations, and provide continuous services, all of which are critical in long-term care settings.

In contrast, financing history and patent counts display more nuanced and, at higher levels, predominantly negative associations with creditworthiness. The model indicates that frequent or complex financing activities may signal financial stress or overreliance on external capital, increasing concerns about long-term solvency. Similarly, intensive patenting activity, when not accompanied by visible improvements in service capacity or governance, may be interpreted as symbolic or speculative innovation rather than substantive enhancement of care quality. Together, these patterns suggest a sector-specific “innovation–stability paradox,” in which strategies aimed at rapid expansion or visible innovation can, paradoxically, increase operational vulnerability and perceived risk.

To translate these analytical findings into practical decision support, the optimized SMOTE- XGBoost model was deployed as a user-friendly web application. By allowing users to input observable institutional characteristics and receive real-time creditworthiness assessments, the tool operationalizes the four key determinants identified above. It thus provides regulators with a scalable instrument for targeted supervision, offers managers a diagnostic tool for self-assessment and risk management, and helps older adults and their families reduce information asymmetry when selecting private long-term care institutions.

### 5.2 Comparison with existing literatures

This study extends prior research in three important respects. First, whereas most evaluations of long-term care institutions rely on subjective measures such as resident satisfaction surveys or qualitative interviews (11, 13), this study demonstrates the feasibility of objective, data-driven assessments using publicly available information. By shifting the focus from perceptual evaluations to institutional-level indicators, it provides a more comprehensive and transparent framework for assessing sustainability in the long-term care sector.

Second, methodologically, the study adapts credit risk assessment models—traditionally applied to individuals and small enterprises (14, 15)—to the context of private long-term care institutions. The integration of class-imbalance handling strategies with ensemble machine learning enhances predictive robustness, as evidenced by the superior performance of the SMOTE- XGBoost combination (AUC = 0.993, with simultaneously high sensitivity and specificity). At the same time, the use of SHAP analysis directly addresses the “black-box” limitations of AI models by revealing how individual features contribute to the predicted creditworthiness scores. This approach aligns with recent calls for interpretable machine learning in healthcare and social governance (33), and our results provide a concrete example of how such methods can be deployed in a new domain while preserving both accuracy and explainability.

Third, beyond methodological contributions, the study adds nuance to the discussion of institutional creditworthiness by identifying four critical determinants: registered capital, workforce size, financing history, and patent counts. Registered capital has long been regarded as a proxy for financial robustness in both corporate risk assessment and social service provision, with prior studies highlighting its role in reducing default risk and ensuring service continuity (19, 26). Our findings corroborate this view, showing a consistent positive relationship between registered capital and institutional creditworthiness. Workforce size, measured by the number of tax-paying employees, reflects both organizational capacity and regulatory compliance. Consistent with evidence from healthcare and governance studies (42), a larger and formalized workforce signals stronger service capacity and enhances credibility.

In contrast, financing history demonstrates a more complex association. While access to financing is often linked to growth potential in small enterprises (17), our results suggest that frequent or complex financing trajectories may undermine institutional stability. This finding is consistent with financial risk literature that emphasizes how overleveraging and unstable capital structures elevate long-term risk (43, 44). Similarly, patent counts highlight a sector-specific “innovation–stability paradox.” Although innovation is typically associated with competitiveness (45, 46), excessive reliance on patents without corresponding improvements in governance or service quality may destabilize operations—a concern echoed by (12).

Together, these findings refine the understanding of institutional creditworthiness in the long-term care sector by demonstrating that financial robustness and human capital investment strengthen credibility, while unchecked financial complexity and symbolic innovation may compromise sustainability.

### 5.3 Policy and practical implications

The results of this study carry important implications for regulatory governance, institutional management, family decision-making, and the broader development of the long-term care sector.

First, for regulators, the proposed framework offers a scalable and cost-efficient tool for strengthening risk-based oversight. In the Chinese context, where supervisory resources are limited relative to the rapid expansion of private long-term care institutions, predictive modeling can help civil affairs departments and other relevant authorities prioritize inspections, focus on facilities with higher predicted risk, and design early warning lists. By continuously updating the model with new data, regulators can move from ex post, complaint-driven supervision toward more proactive, data-driven risk monitoring, thereby reducing blind spots in inspections and enhancing consumer protection.

Second, for institutional managers, the identification of key determinants provides concrete guidance for strategic and operational decisions. The positive roles of registered capital and workforce size suggest that managers should pay sustained attention to capitalization adequacy, long-term investment in staff, and formal employment arrangements, rather than relying solely on short-term cost-cutting. Conversely, the negative associations of complex financing histories and excessive patenting highlight the risks of pursuing rapid, leveraged expansion or symbolic innovation without parallel improvements in governance and service quality. In practical terms, managers should adopt conservative financing policies, strengthen internal risk management and disclosure, and embed innovation within a broader framework of stable operations and quality assurance to avoid falling into the “innovation–stability paradox.”

Third, for families and service users, the web-based application helps address the pronounced information asymmetry that characterizes China’s long-term care market. Many families, especially those caring for frail or cognitively impaired older adults, lack the expertise and time to collect and interpret institutional information. By translating complex risk models into intuitive risk levels and visual outputs, the tool provides an accessible reference that can be used alongside on-site visits and word-of-mouth evaluations. This not only supports more informed and transparent choices of institutions, but also enhances the bargaining power and confidence of families in interactions with service providers. This transparency function is particularly valuable for vulnerable households facing urgent placement decisions.

Finally, for the long-term care sector as a whole, the integration of interpretable AI into governance practice illustrates how digital technologies can promote transparency, accountability, and resilience in the era of the silver economy. At the system level, such tools can be embedded into local or national credit information platforms, linked with licensing, subsidy allocation, or performance evaluation mechanisms, and used to stimulate a virtuous cycle in which high-performing institutions are rewarded and underperforming ones are identified and rectified more promptly. In this way, the framework contributes not only to micro-level decision-making but also to the construction of a more sustainable, trustworthy, and competitive institutional care market.

### 5.4 Limitations and future research

Several limitations should be acknowledged. First, the dataset is limited to institutions in Hangzhou, which may constrain the generalizability of findings. Future studies should expand the geographical scope to capture regional variation in regulatory frameworks and market conditions. Second, the dependent variable of creditworthiness is based on third-party ratings, which may contain measurement bias or omit qualitative aspects of care quality. Combining institutional-level indicators with government inspection records or resident-reported outcomes would enhance validity. Future work could triangulate labels using multiple sources (e.g., administrative compliance records, subsidy eligibility, or documented service interruptions) to reduce platform-specific measurement bias. Third, the current framework focuses on structural and financial attributes. Incorporating additional dimensions, such as service quality, staffing competencies, and user satisfaction, could provide a more holistic assessment of institutional sustainability.

## 6 Conclusion

This study developed a novel credit risk assessment framework for private long-term care institutions in China, addressing the urgent need for transparent and objective evaluation mechanisms under the silver economy. Using legally collected public data from 594 institutions in Hangzhou, the framework integrated class-imbalance handling techniques with machine learning models. The XGBoost model combined with SMOTE resampling demonstrated superior performance, achieving both high discriminative power and robust generalizability.

Beyond predictive accuracy, the study employed feature importance and SHAP analysis to enhance interpretability, identifying four key determinants of institutional creditworthiness: registered capital, number of tax-paying employees, financing history, and number of patents. While the first two contribute positively to financial stability and organizational resilience, the latter two revealed negative associations at higher levels, reflecting an “innovation–stability paradox” unique to the long-term care sector.

The contributions of this research are threefold. First, it extends credit risk methodologies to the long-term care domain, enriching the literature with institution-level, data-driven evidence. Second, it demonstrates the value of interpretable machine learning in uncovering the mechanisms that shape institutional sustainability. Third, it bridges methodological innovation and practice by deploying a publicly accessible web tool, thereby supporting regulators, managers, and families in evidence-based decision-making.

Looking ahead, expanding the framework to cover broader geographical regions, incorporating additional qualitative dimensions of care quality, and integrating multiple data sources will further enhance its robustness and applicability. Collectively, the study offers a data-driven pathway toward greater transparency, accountability, and resilience in long-term care provision, contributing to the sustainable development of the sector in the era of the silver economy.

## Data Availability

The raw data supporting the conclusions of this article will be made available by the authors, without undue reservation.

## Footnotes

1
https://www.tianyancha.com/
