# Supplementary material for: Credit risks in long-term care: a novel assessment of private long-term care institutions for sustainable development under the silver economy in China
Source: Front Public Health. 2026 Apr 29;14:1790055. doi: 10.3389/fpubh.2026.1790055 (PMC13168103; doi:10.3389/fpubh.2026.1790055)
Supplement: Supplementary file 1 [file Table_1.DOC]

## Appendix Table A1. Final hyperparameter settings for the six classifiers

| **Model** | **Final hyperparameter settings** |
| --- | --- |
| CART | criterion = gini; splitter = best; max_depth = None; min_samples_split = 2; min_samples_leaf = 1; max_features = None; class_weight = None |
| C4.5 | criterion = entropy; splitter = best; max_depth = None; min_samples_split = 2; min_samples_leaf = 1; max_features = None; class_weight = None |
| RF | n_estimators = 100; criterion = gini; max_depth = None; min_samples_split = 2; min_samples_leaf = 1; max_features = sqrt; bootstrap = True; random_state = 42 |
| SVM | C = 1.0; kernel = rbf; gamma = scale; probability = True; random_state = 42; class_weight = None; tol = 1e-3 |
| Bagging | n_estimators = 50; base_estimator = None; max_samples = 1.0; max_features = 1.0; bootstrap = True; random_state = 42 |
| XGBoost | n_estimators = 100; max_depth = 6; learning_rate = 0.3; booster = gbtree; subsample = 1.0; colsample_bytree = 1.0; gamma = 0; reg_alpha = 0; reg_lambda = 1; random_state = 42 |

**Appendix Table A2. Additional test-set performance metrics of different models under four sampling strategies**

| Sampling strategy | Metric | CART | C4.5 | RF | SVM | Bagging | Xgboost |
| --- | --- | --- | --- | --- | --- | --- | --- |
| Over-sampling | Precision | 0.523 (0.395-0.671) | 0.489 (0.414-0.692) | 0.600 (0.511-0.781) | 0.477 (0.361-0.637) | 0.556 (0.402-0.684) | 0.659 (0.523-0.786) |
|  | F1 | 0.630 (0.521-0.767) | 0.595 (0.523-0.744) | 0.730 (0.657-0.869) | 0.575 (0.449-0.712) | 0.676 (0.532-0.788) | 0.771 (0.677-0.879) |
|  | PR-AUC | 0.709 (0.647-0.811) | 0.714 (0.623-0.736) | 0.846 (0.831-0.886) | 0.857 (0.831-0.881) | 0.865 (0.701-0.879) | 0.895 (0.838-0.902) |
| Under-sampling | Precision | 0.535 (0.442-0.732) | 0.511 (0.414-0.686) | 0.551 (0.458-0.716) | 0.436 (0.299-0.547) | 0.543 (0.438-0.706) | 0.625 (0.567-0.826) |
|  | F1 | 0.639 (0.581-0.818) | 0.622 (0.567-0.764) | 0.692 (0.628-0.834) | 0.571 (0.426-0.667) | 0.667 (0.591-0.816) | 0.725 (0.712-0.894) |
|  | PR-AUC | 0.711 (0.695-0.832) | 0.725 (0.673-0.802) | 0.832 (0.801-0.882) | 0.846 (0.825-0.936) | 0.851(0.805-0.884) | 0.889 (0.814-0.894) |
| Over+Under sampling | Precision | 0.601 (0.449-0.728) | 0.595 (0.448-0.728) | 0.651 (0.500-0.766) | 0.479 (0.344-0.612) | 0.635 (0.448-0.728) | 0.737 (0.594-0.865) |
|  | F1 | 0.702 (0.568-0.807) | 0.704 (0.568-0.807) | 0.778 (0.657-0.861) | 0.597 (0.459-0.717) | 0.725 (0.568-0.807) | 0.836 (0.732-0.916) |
|  | PR-AUC | 0.761 (0.683-0.824) | 0.762(0.693-0.824) | 0.856(0.845-0.891) | 0.897 (0.821-0.916) | 0.903 (0.892-0.951) | 0.913 (0.887-0.956) |
| SMOTE | Precision | 0.703 (0.546-0.835) | 0.889 (0.800-0.935) | 0.794 (0.634-0.910) | 0.564 (0.418-0.694) | 0.703 (0.546-0.835) | 0.824 (0.688-0.929) |
|  | F1 | 0.788 (0.662-0.877) | 0.857 (0.836-0.980) | 0.857 (0.745-0.937) | 0.647 (0.508-0.757) | 0.788 (0.662-0.877) | 0.889 (0.796-0.956) |
|  | PR-AUC | 0.815 (0.726-0.855) | 0.819 (0.798-0.895) | 0.874 (0.762-0.896) | 0.905 (0.826-0.931) | 0.954 (0.912-0.971) | 0.983 (0.971-0.991) |

Note: Values are point estimates on the held-out test set; values in parentheses are 95% confidence intervals. PR-AUC denotes the area under the precision-recall curve. Source: Authors’ own computation.

Appendix Table A3. Training-set classification performance of different models under four sampling strategies

| **Sampling strategy** | **Metric** | **CART** | **C4.5** | **RF** | **SVM** | **Bagging** | **Xgboost** |
| --- | --- | --- | --- | --- | --- | --- | --- |
| Over-sampling | Sensitivity | 0.892 (0.716-0.912) | 0.964 (0.911-0.976) | 0.982 (0.972-0.991) | 0.940 (0.917-0.967) | 0.952 (0.934-0.978) | 0.964 (0.918-0.978) |
|  | Specificity | 0.953 (0.913-0.987) | 0.925 (0.910-0.956) | 0.983 (0.917-0.993) | 0.912 (0.897-0.932) | 0.983 (0.925-0.991) | 0.982 (0.967-0.992) |
|  | Accuracy | 0.942 (0.937-0.977) | 0.933 (0.912-0.967) | 0.984 (0.921-0.991) | 0.917 (0.876-0.933) | 0.978 (0.911-0.988) | 0.977 (0.915-0.982) |
|  | AUC | 0.922 (0.867-0.935) | 0.945 (0.878-0.976) | 0.995 (0.886-0.999) | 0.977 (0.965-0.991) | 0.995 (0.919-0.999) | 0.995 (0.919-0.999) |
| Under-sampling | Sensitivity | 0.711 (0.661-0.788) | 0.759 (0.678-0.801) | 0.807 (0.771-0.910) | 0.904 (0.854-0.932) | 0.759 (0.712-0.832) | 0.759 (0.701-0.776) |
|  | Specificity | 0.691 (0.561-0.789) | 0.804 (0.712-0.854) | 0.829 (0.789-0.856) | 0.812 (0.756-0.826) | 0.873 (0.842-0.901) | 0.823 (0.801-0.865) |
|  | Accuracy | 0.694 (0.598-0.765) | 0.796 (0.714-0.823) | 0.825 (0.756-0.865) | 0.829 (0.801-0.865) | 0.852 (0.799-0.888) | 0.811 (0.789-0.856) |
|  | AUC | 0.701 (0.655-0.742) | 0.781 (0.712-0.823) | 0.911 (0.877-0.945) | 0.932 (0.901-0.966) | 0.905 (0.887-0.933) | 0.885 (0.845-0.902) |
| Over+Under sampling | Sensitivity | 0.952 (0.845-0.966) | 0.952 (0.902-0.977) | 0.976 (0.931-0.988) | 0.964 (0.915-0.987) | 0.976 (0.902-0.986) | 0.988 (0.931-0.991) |
|  | Specificity | 0.890 (0.756-0.923) | 0.892 (0.801-0.936) | 0.859 (0.821-0.897) | 0.878 (0.806-0.921) | 0.870 (0.822-0.936) | 0.909 (0.877-0.912) |
|  | Accuracy | 0.901 (0.874-0.932) | 0.903 (0.888-0.923) | 0.881 (0.855-0.903) | 0.894 (0.875-0.936) | 0.890 (0.779-0.932) | 0.924 (0.885-0.945) |
|  | AUC | 0.921 (0.885-0.936) | 0.922 (0.887-0.945) | 0.975 (0.883-0.991) | 0.974 (0.922-0.983) | 0.969 (0.945-0.993) | 0.988 (0.906-0.993) |
| SMOTE | Sensitivity | 0.952 (0.906-0.988) | 0.928 (0.903-0.966) | 0.976 (0.913-0.986) | 0.976 (0.903-0.986) | 0.988 (0.906-0.992) | 0.988 (0.883-0.993) |
|  | Specificity | 0.942 (0.906-0.978) | 0.950 (0.889-0.988) | 0.934 (0.896-0.965) | 0.948 (0.863-0.978) | 0.939 (0.877-0.965) | 0.967 (0.912-0.988) |
|  | Accuracy | 0.944 (0.899-0.967) | 0.946 (0.875-0.987) | 0.942 (0.879-0.963) | 0.953 (0.912-0.966) | 0.951 (0.909-0.988) | 0.971 (0.899-0.989) |
|  | AUC | 0.947 (0.896-0.977) | 0.939 (0.877-0.977) | 0.994 (0.888-0.999) | 0.985 (0.921-0.991) | 0.994 (0.901-0.998) | 0.996 (0.921-0.999) |

Note: Values are point estimates on the resampled training data; values in parentheses are 95% confidence intervals. These results are provided to facilitate comparison with the held-out test-set performance reported in Table 3.

Appendix Table A4. Variance inflation factor (VIF) values for explanatory variables

| **Variable code** | **VIF** | **Variable code** | **VIF** |
| --- | --- | --- | --- |
| CS06 | 1.051 | CS03 | 3.487 |
| CS04 | 1.357 | CR05 | 3.680 |
| CP04 | 1.365 | CW01 | 3.780 |
| CP02 | 1.435 | CP03 | 3.854 |
| CR03 | 1.565 | CP05 | 4.720 |
| CR04 | 1.701 | CW03 | 4.818 |
| CR02 | 1.723 | CS02 | 4.850 |
| CP01 | 1.937 | CS05 | 5.691 |
| CS01 | 2.475 | CR01 | 6.385 |
| CW04 | 3.196 | CW02 | 6.439 |
